# 

**Published:** 1830-06

**Authors:** 


### MONTHLY LIST OF MEDICAL BOOKS.

[*Medical Works cannot be entered on this List except a copy be sent for the purpose; the titles of Books having frequently been transmitted to us, as published, which have not appeared for weeks, or even months, after.*]

On the Diseases and Injuries of Arteries, with the Operations required for their Cure: being the Substance of the Lectures delivered in the Theatre of the Royal College of Surgeons, in the Spring of 1829. By G. J. GUTHRIE, F.R.S. &c.—8vo. pp. 416. Burgess and Hill, London, 1830.

A Descriptive Road-book of Germany. By E. A. DOMEIER, B.A. and M.B. —Leigh, Strand, 1830.

The best-arranged guide for the traveller to Germany that has yet appeared. It is furnished with a very copious index, and contains a list of the German universities; specimens of two of the chief dialects; and a catalogue of plants which are not given in any other English book.

Modern Medicine influenced by Morbid Anatomy; An Oration delivered at the 57th Anniversary of the Medical Society of London. Also, an Apology for Medical Nomenclature. By LEONARD STEWART, M.D. &c.—8vo. pp. 56. Longman, London, 1830.

Remarks on Nervous and Mental Disorder, with especial Reference to recent Investigations on the Subject of Insanity. By DAVID UWINS, M.D. —Pp. 41. Underwood, 1830.

A Popular Description of the Aldinian Defensive Dresses, &c. for rescuing Human Life and Property from Injury or Destruction in Cases of Fire.—Pp. 24. Ridgway, London.

Cholera, its Nature, Cause, and Treatment; with original Views, physiological, pathological, and therapeutical, in relation to Fever; the Action of Poisons on the System, &c. To which is added, an Essay on Vital Temperature and Nervous Energy; explanatory, more particularly, of the Nature, Source, and Distribution of the latter, and of the Connexion between the Mind and the Body, &c. By CHARLES SEARLE, Surgeon, of the Hon. East-India Company's Madras Establishment.—8vo. pp. 255. Wilson, London, 1830.

### INDEX

TO THE

EIGHTH VOLUME OF THE NEW SERIES

OF THE

### London Medical and Physical Journal.

PAGE

Abortion caused by mercury 300
Abscess communicating with the caput coli . . . 265
Ague, ergot in . . . 556
—— cured by charcoal . 365
Air introduced into the veins of a cock . . . 183
Allantoid, dropsy of . . 275
Amaurosis from diseased tooth 464
—— probably occasioned by irritation of the medulla spinalis . . . 99
Amenorrhœa caused by mercury 300
Ammonia, violent inflammation from inhalation of . . 515
Amnion, on the "false waters" of the . . . . . 459
Amnii, incessant discharge of liquor, previous to labour, four months . . . 115
Anatomical Museum at Guy's, catalogue of . . . 544
Aneurism of the aorta; rupture into the trachea . . 49
——, both carotids tied in a successful case . . 482
—— of carotid from a blow 129
—— of right subclavian artery . . . . . 285
Angina pectoris, connected with cervical tenderness . . 214
Ani, prolapsus, treated as by Hey, 557
Animal acids, poisonous properties of . . . 528
—— matter, poisoning by decayed . . . 530
—— ——, poisonous by putrefaction . . . 531
Aorta, aneurism of; rupture into the trachea . . . 49
Apoplexy, 9 cases in 63 with precursory symptoms . . 155

PAGE

Apothecaries, new regulations of the Society of . . 372
Armstrong, Dr. death of . 91
Arsenic, very large doses given in lepra . . . 272
Asparagus, sedative action of . 276
Asphyxia from charcoal vapour; recovery . . . 495
Asthma, remarks on nervous . 311

Belladonna locally applied in retention of urine from stricture 326
—— in frontal neuralgia 317
Bile mixed with the blood: does it prevent coagulation? . 502
Bladder, severe wound of . 367
Blisters, cotton as a dressing for 83
Blood, remote effects of loss of 169
——, on the morbid and curative effects of loss of . 167, 226
——, tolerance or intolerance of loss of . . . 189
——, effects of loss of, resembling increased power . 168
——, remarks on the . 562
——, treatment of the effects of loss of . . . 171
——, impure state of, from failure in secretion of urine . 487
Bloodletting, observations on . 189
Botanical lectures, Mr. G. Burnett's . . . 374
Botany, Forsyth's "First Lines" of 72
Brain, functions of suspended, from irritation of the stomach 361
—— affected by non-secretion of urine or perspiration . 488
Breast, cases of open cancer of, by H. Mayo, F.R.S. . . 10
——, cases of Scirrhus, by H. Mayo, F.R.S. . . . 1
Bronchocele, use of iodine in 63

PAGE
Bronchocele, case of, treated by iodine . . . 480
———— relieved by iodine 464
Brucia, use of, in paralysis . 59
Buccina, new principle in box-wood . . . 467
Burrows, Letter to Dr. G. M. . 568
————, Dr. Letter to Sir H. Halford . . . 427
————, why attacked in the Quarterly Review . 433

Calculous diseases, supposed reasons for the frequency of, in Norfolk . . . 445
Camphor, on effects of, in health 466
Cancer aquaticus, chlorate of lime used in . . . 561
Carotid arteries, both, tied with success . . . 482
———— artery, aneurism of, from a blow . . . 129
———— tied for hemorrhage from sloughing of the throat . . . 85
Cerebellum, influence of, over generative faculties . . 77
Chabert, M. his experiments as a poison swallower . . 281
Charcoal, recovery from exposure to vapour of . . . 495
———— in ague . . 365
Chorea, remedies in . . 64
Climate, notice of Dr. Clark's work on the Influence of . 471
Cloquet's Anatomy, translation of, by Mr. King . . 472
Colchicum, use of, in dropsy and rheumatism . . . 61
Cold affusion; how it acts . 69
Colica pictonum, pathology of 82
————, morbid appearances in . . . 526
————, prophylactic measures against . . 527
Colon, on distention of . 441
———— stricture of . . 123
Coma from irritation of the spinal cord . . . . 105
Convulsions from excessive drinking . . . 389
Combustion, case of spontaneous 360
Copper, poisoning by . . 521
Corpora lutea, on the formation of the . . . . 333
Cotton as a dressing for blisters 83
Cough from irritation of the spinal cord . . . . 307
Counter-irritants, remarks on . 195
Cranium, on affections of bones of, 458

PAGE
Croup, irritation of nerves producing spasmodic . . 314
————, sulphate of copper in . 556
————, on spasmodic . . 380
————————, in infants 384
Cuticle, disorganization of the, in infants . . . 265

Darnel grass, poisoning with . 542
Datura Stramonium, poisoning with . . . . 538
Delirium, on the efficacy of anodyne clysters in traumatic . 287
————, opium freely used in 112
———— from spinal affections 102
———— Tremens, large doses of opium given in traumatic 503
————, morbid appearances in a case of . 365
————; result of different treatment in . . 84
Delivery, on the causes and prevention of premature . 299
Delphia, inefficacy of, as a remedy in disease . . 53
Diabetes, remedies in : . 64
Diarrhœa, use of strychnia in chronic . . . 58
Digitalis, use of, in hydrocephalus . . . 83
————, death from infusion of 539
Diploma, medical, from London University . . . 566
Double female infant, post-mortem examination of . . 89
Drinking, convulsions from excessive . . . 389
Dropsy, colchicum in cases of 61
Dulcamara, on the uses and properties of . . . 393
Duodenum, rupture of, resembling effects of poison . 457
Duplex child, case of . . 468
Dyspepsia, use of gentiana in . 63

Elbow-joint, on excision of . 421
Emetina superior to ipecacuanha 63
Epilepsy, on the treatment of . 463
Ergot of rye produces abortion in animals . . . 84
————, analysis of . 371
Eyes, efficacy of lemon-juice in some diseases of . . 366
Eye, nitrate of silver used in diseases of . . 219, 275
——, use of turpentine in inflammation, &c. of the . . 47
——, efficacy of turpentine in some diseases of the . . 320
——, diseases of . . 219

PAGE
Face, Reply of a Pupil to some remarks in the Critical Analysis on Mr. C. Bell's Paper on the Nerves of the . . 27
Fever, external use of opium in 70
——, Dr. S. Smith on . 234
——, on the nature of . 65
——, remittent, caused by an abscess near the anus . 174
—— sometimes destroys instantaneously . . . 254
——, treatment of . . 256
——, "cold dash" in . . 259
——, how it differs from inflammation . . . 229
——, Dr. Tweedie on . 234
——, errors of theorists on . 237
——, essence of, erroneously attributed to a morbid condition of the blood . . 237
—— not solely caused by inflammation of the brain . ib.
——, identity of, under different circumstances . . 239
—— not an entity, but a series of events . . . 240
—— compared with inflammation . . . 241, 255
——, various parts affected in 242
——, diversity of symptoms of, from different degrees of affection of the same organs . 243
——, on simple . . 245
——, continued, only one disease, of different aspects and intensities . . . ib.
——, changes of structure in, without pain . . 247
——, affections of brain during ib.
——, remarks on typhous . 249
——, pathology of . . 253
——, fatality of, during pregnancy . . . ib.
Fires, hints for the suppression of 377
Fish, species which are deleterious . . . . 529
Fistula, vesico-vaginal, cured by actual cautery . . 510
—— in ano opening into the rectum . . . 426
——, parotid, cured by sulphuric acid . . . 26
Fœtus, on the causes and prevention of the death of the . 299
——, rudiments of, found in the testicle of an infant . 358
Fractures, M. Larrey's plan of treating . . . 465
Functional disease, import of the term . . . . 93

PAGE
Fungus hæmatodes treated with pyroligneous acid . . 418

Gangrene treated with pyroligneous acid . . . ib.
Gases, on the poisonous . 535
Gastritis, effects of, in infants . 80
General practitioners allowed to charge for *attendance* . 183
Generative faculties, influence of the cerebellum on . . 77
Genitals, on diseases of the male 347
Gentiana, use of, in dyspepsia . 63
Gestation, Dr. Granville on protracted . . . 89
Gonorrhœa and syphilis, poisons of, the same . . 552
—— cured by copaiba injections . . . 275
Gonorrhœal ophthalmia, treatment of . . . 84
Gooch, death of Dr. . . 283
Guthrie, G. J. F.R.S., his instrument for breaking stones in the bladder . . . 564

Hæmatemesis, utility of ipecacuanha in . . . 272
Handey *v.* Henson; trial in the King's Bench, by which it was decreed that a general practitioner can legally charge for *attendance* . . . 185
Head, severe injury of . . 110
——, affections of, from irritation of cervical portion of the spinal cord . . . 95
Heart, wound of the . . 177
——, ossification of its valves after rheumatism . . 323
——, disease of, after rheumatism . . . . ib.
——, simulated, from compression of the phrenic and pneumo-gastric nerves . 365
Hernia, use of turpentine in strangulated . . . 401
——, umbilical, treated by ligature . . . 559
——, incarcerated, internal use of Sp. Terebinth. in . 217
Heat produced by the functions of animal life . . 345
Hooping cough, treatment of . 365
——, morphia externally applied in . . 274
Horse-radish, utility of, as a remedy in different diseases . 370
Hunterian oration, opinion of Mr. Guthrie's . . 280

PAGE

Hydrocephalus, tapping successful in . . . 415
————, use of digitalis in 83
Hydrocyanic acid, on the presence of, in the human body 468
————, poisoning with 533
————, symptoms caused by, in man . . 534
Hydrophobia, symptoms resembling, from spinal irritation . 386
————, experiments, &c. relating to . . . 460
———— from cold . 81
————, chlorine in . 275
Hysteria arising from functional disorder of the spinal cord . 13

Ileus, cases of simple . . 553
Indigestion causing mania . 82
Insects, how to be killed for cabinets . . . 276
Intestine, internal strangulation of, 416
————, resembling the effects of poison . 418
Iodine, use of, in bronchocele, scrofula, dropsy, &c. . 63
————, effects of, on the genitals 464
————, passage of, into the blood 181
———— in scrofula . . 176
Ipecacuanha, utility of, in hæmatemesis . . . 272
————, emetina superior to 63
Iris, congenital absence of . 78
Irritation, remarks on . . 231
Irritants, remarks on counter 195

Jaundice, cases of, with diseased pancreas . . . 499
Joints, on excision of the elbow and knee . . . 421
————, on removal of diseased 368

Kidneys, functional disorders of 444
————, paralysis of . . 484
Knee-joint, on excision of . 421

Labour retarded by preternatural septum in vagina . 177
Lactation, curious metastasis during . . . 461
Laryngotomy, successful case of 176
Lead, on poisoning by . . 523
————, tests of . . . ib.
Leech, mechanical . . 183
Leeches, application of, to infants reprobated . . 234
Lion, prickle in the tail of the 182
Lister, Dr. W., biographical sketch of . . . 469

PAGE

Lithotrity, description of Baron Heurteloup's instruments . 489
————, Mr. Lukin's instruments 494
Liver, abscess of, communicating with duodenum . . 79
Lolium temulentum, poisoning with . . . . 542
London University, prizes distributed at . . . 564
Lues venerea not a specific disease . . . . 351
Lupulia, inefficacy of, as a remedy in disease . . 53

Macmichael, Dr. appointed librarian to his Majesty . . 469
Mania from indigestion . 82
Medical Provident Institution of Scotland . . . 282
Menorrhagia produced by mercury . . . . 300
Mercurial erethism . . 519
Mercury, meagre diet prejudicial during use of . . 356
————, poisoning by . 517
————, a *cumulative* poison . 519
———— recommended to prevent premature delivery and death of the fœtus . . 300
Midwifery report by Mr. Waller 120
Milk, on suspending the secretion of . . . 462
Mole, on the vision of the . 88
Morbid Anatomy, Vade-mecum of . . . . 450
Morphia, acetate of, in neuralgia 131
————, use of acetate of, in gastralgia . . . 60
———— in neuralgia 61
———— in scirrhus and induration of uterus . 60
Mortality, Bills of . . 187
Motion remaining, sensation being destroyed . . 359

Nerves of the face; opinions of Mr. Bell and Mr. Mayo respecting . . . 45
————. Reply of "A Pupil" to remarks in the Critical Analysis on Mr. C. Bell's paper on the . . 27
————, effects of dividing pneumo-gastric . . . 77
————, affections of origins of, indicated by pain at minute extremities . . . 16
Nervous system and other parts, proportion between the . 358

PAGE
Nervous system; facts proving that it regulates the developement of the embryo . . 358
—— action, on . . 65
—— diseases; their connexion with functional disorders of the spinal cord . 13, 93, 207, 307
Neuralgia, use of morphia in . 61
——, acetate of morphia in 131
——, on external use of belladonna in frontal . . 317

Oak, Mr. G. Burnett on natural history of . . . 280
Œsophagus, foreign bodies extracted from . . . 370
Opium, external use, in fever . 70
——, treatment of poisoning with . . . . 532
Oppression arising from spinal irritation . . . 311
Otitis, remarks on . . 291
Ovaria, diseases in the structure of . . . 334
——, treatment of diseases of 339
——, remarks on extirpation of diseased . . . 341
——, on diseases of . . 329
——, functions of, in various animals . . . . ib.
Ovarium, one wanting . 334
Ova seen in the Fallopian tubes 332
Oxalic acid, periods when fatal 513
——, cases of poisoning by (*note*) . . . 514

Pancreas diseased in jaundice . 499
Paralysis in a child, with anomalous symptoms . . 363
——, use of strychnia in . 53
—— brucia in . 59
—— of face cured by galvanism . . . 463
—— cured by strychnia . 130
Parotid fistula cured by sulphuric acid . . . 26
—— gland, extirpation of . 370
Penis, diseases of . . 351
Phlegmasia dolens, with symptoms of phlebitis . . 267
Phlebitis, uterine, with purulent effusion in knee-joint . . 223
—— after parturition . 224
Phosphorus swallowed in large quantities . . . 374
Phthisis, remarks on, showing the advantages of free exposure to the air, &c. . . . 405
—— from irritation of respiratory nerves . . . 308

PAGE
Phthisis, use of acetate of lead in 464
Physics, on the Elements of . 343
Picrotoxia, inefficacy of, in disease . . . . . 53
Piles, remarks on . . 442
Placenta, morbid adhesion of, to uterus . . . 121
——, ergot of rye effecting expulsion of retained . 123
——, ossification in the . 178
—— and uterus, circulation between the . . . 452
Pneumo-gastric nerves, effects of their division . . 77
Poisons, absorbed; or effects of transmitted by sentient extremities of nerves . . 140
——, review of treatises on . 133
——, evidence of, from symptoms . . . . 151
—— —— morbid appearances doubtful . 157
—— —— chemical analysis . . . 161
—— —— experiments on animals . . 163
—— —— from moral circumstances . . 165
Poisons, effects of, compared with some natural diseases . 153
——, symptoms of narcotic 155
——, irritant . . 511
——, local effects of . 454
Polypus of vagina removed by local use of laudanum . 369
Potass, sulphuret of, poisoning by 515
Prophetic power before death in brain fever . . . 277
Prostate gland, case of abscess in . . . . . 462
—— —— and rectum, extensive disease of . . 266
Prussic acid, M. Chabert's "antidote" for . . . 374
Pulmonary tubercles, on . 175
Pyroligneous acid used in gangrene, &c. . . . 418

Rectum, severe wound of . 367
——, on stricture of . 435
Respiration, on the means of affording, to children, in reversed presentations . . 178
Rheumatic gout, ossification of the valves of the heart after . 322
Rheumatism, colchicum in cases of 61
—— followed by disease of the heart . . . 323

Salivation from various causes . 518

PAGE

Salivation, mercurial, checked by Ant. Tart. . . 521
Savine applied to a fresh wound produces inflammation . 527
Secale cornutum, poisonous effects of . . . 541
Secretion under the *influence* of the nervous system . . 67
Sensation lost, motion remaining 359
Scrofula, iodine in . 63, 176
Soemmering, S. T. death of . 471
Solania, inefficacy of, as a remedy in disease . . 53
Sorbus acuparia, spirit from . 180
Spinal irritation producing symptoms like hydrophobia . 386
————, case of, with transference of the diseased action from one organ to another . . . . 387
Spinal cord, functional disorders of . . . . . 379
————, diseases of, confounded with poisons . 156
————, irritation of, inducing affections of the head, neck, chest, stomach, and extremities 95
————, Dr. and Mr. D. Griffin on functional disorders of, and their connexion with hysteria, &c. 13, 93, 207, 307
————, on irritation of . 23
————, functional disorder of, resembling inflammation of the viscera . . . . 14
Spirit, new source of . . 180
Stammering, nature and cure of 342
———— rare in women . 79
Stomach, distention of, resembling effects of poison . 455
————, rupture of, without evident cause . . 475
————, perforation of 79, 215
————, rupture of, resembling effects of poison . . 456
————, use of morphia in painful affections of . . 60
————, irritation of, causing suspension of the cerebral functions . . . 361
Stramonium in retention of urine 367
Stricture causing retention of urine, and relieved by local application of belladonna . 326
Strychnia, use of, in paralysis . 53
———— diarrhœa . 58
————, poisoning by . 276
————, rapidly fatal effects of 540
————, utility of, in paralysis 130

PAGE

Syphilis treated with salt . 84
———— and gonorrhœa, poisons of, the same . . . 552
———— known from a very early period . . . 348
————, comparative frequency of secondary symptoms of, after various modes of treatment 349
————, mercury not a specific remedy for . . . 351

Tallow, poisoning by slow combustion of . . . 537
Tapping in hydrocephalus successful . . . 415
Throat, carotid artery tied for sloughing of . . . 85
Tongue, diagnostic appearances of . . . . 454
Tonsils, on excision of . . 465
Tormentilla, use of decoction of, for warts . . . 464
Touch, on the sense of . 358
Tubercles in the lungs of an infant, 174
————, on pulmonary . 175
Turpentine, use of, in strangulated hernia . . . 401
————, efficacy of, in some diseases of the eye . . 320
————, use of spirits of, in incarcerated hernia . . 217
Twins united by the umbilicus 453

Ulcers treated with pyroligneous acid . . . . 418
Ulna, luxation of, backwards . 327
Umbilical hernia treated by ligature . . . . 559
Urine, datura stramonium, in retention of . . . 367
————, retention of, from stricture; belladonna successfully applied . . . 326
Uterine phlebitis, with ulceration of the articular cartilage, and purulent effusion in the knee-joint . . . 223
Utero-gestation, protracted . 467
Uterus and placenta, circulation between the . . . 452
————, case of retroverted; uterus punctured with success . 507
————, use of morphia in scirrhus and induration of . . 60
————, pathological researches on inflammation of veins of . 267
———— affected from stricture in the rectum . . . 438
Uterus, dropsy of the gravid . 459

PAGE

Vaccination, summary of . 447
————; why eighth day vesicle to be preferred . 449
Vagina affected from stricture in the rectum . . . 438
———, labour retarded by preternatural septum in . 177
Varicose veins, fatal hemorrhage from . . . . 264
Vegetable acrids, poisonous properties of . . . 527
———— physiology, curious phenomenon in . . . 181
Veins, air introduced into the . 183
Veratria, use of, in dropsy and rheumatism . . . 61
Vertebræ, successful case of luxation of cervical . . 477
————, whether luxated in hanging . . . 480
Vision of the mole . . 88
——— affected by irritation of the spinal cord . . 98

Warts cured by Dec. Torment. 464
Water-cresses, dangerous plant growing among . . 182

### CRITICAL ANALYSES.

Hospital Facts and Observations, illustrative of the Efficacy of the new Remedies, Strychnia, Brucia, Acetate of Morphia, Veratria, Iodine, &c. in several morbid Conditions of the System. By J. L. BARDSLEY, M.D. . . . . . . . . . . . . 52
Notions on the Nature of Fever and of Nervous Action. By W. F. BOW, M.D. . . . . . . . . . . . 65
The First Lines of Botany. By J. S. FORSYTH, Surgeon . . 72
A Treatise on Poisons, in relation to Medical Jurisprudence, Physiology, and the Practice of Physic. By R. CHRISTISON, M.D. . 133, 511
Die Lehre von den Giften, in Medizinischer, Gerichtlicher, und Polizeylicher Hinsicht. Von Dr. K. F. MARX . . . . . 133
An Essay on the Operation of Poisonous Agents upon the Living Body. By JOHN MORGAN, F.L.S. and THOMAS ADDISON, M.D. . . ib.
Researches principally relative to the Morbid and Curative Effects of Loss of Blood. By MARSHALL HALL, M.D. F.R.S.E. &c. . 167, 226
A Treatise on Fever. By SOUTHWOOD SMITH, M.D. . . . . 234
Clinical Illustrations of Fever; comprising a Report of the Cases treated at the London Fever Hospital, 1828-1829. By ALEX. TWEEDIE, M.D. ib.
Illustrations of some of the principal Diseases of the Ovaria, their Symptoms and Treatment. By E. J. SEYMOUR, M.D. . . . 329
Elements of Physics; or Natural Philosophy, General and Medical. Vol. II. Part I.; comprehending the Subjects of Heat and Light. By NEIL ARNOTT, M.D. . . . . . . . . . . 342
A Practical Treatise on Diseases of the Genitals of the Male. By J. M. TITLEY, M.D. . . . . . . . . . . 347
A Letter to Sir H. HALFORD, Bart. K.C.H. &c., touching some Points of the Evidence, and Observations of Counsel, on a Commission of Lunacy on Mr. Edward Davies. By G. M. BURROWS, M.D. . . 427
A Practical Essay on Stricture of the Rectum. By F. SALMON. Third Edition . . . . . . . . . . . . 434
Observations on the Functional Disorders of the Kidneys which give rise to the Formation of Urinary Calculi. By W. ENGLAND, M.D. . 444
A Popular Summary of Vaccination. By JOHN MARSHALL, Esq. . 447
A Vade-Mecum of Morbid Anatomy, Medical and Chirurgical . 450
A Catalogue of the Preparations in the Anatomical Museum of Guy's Hospital. Arranged and edited by THOMAS HODGKIN, M.D. . 544

### NAMES OF AUTHORS,

*Of whose Works, Observations, &c. either a detailed Account, or more or less general View, is given in the present Volume.*

PAGE

ABERCROMBIE, Dr. case of Diseased Rectum and Prostate Gland . 266
——————— cases of Ileus . . . . . . 553
ADDISON, Dr. T. review of his Essay on the Operation of Poisonous Agents upon the living Body . . . . . 133
ANGUETIN, Dr. N. P. on Colica Pictonum . . . . . 82
ALBERT, Dr. case of Protracted Utero-gestation . . . 467
ALCOCK, THOMAS, esq. notice of his Lectures on Practical and Medical Surgery . . . . . . . . . 472
ALIX, M. on a curious Phenomenon in Vegetable Physiology . 181
ARNOTT, Dr. NEIL, Elements of Physics (review) . . . 342

BARDSLEY, Dr. J. L. review of his "Hospital Facts," &c. . . 52
——————— case of Paralysis cured by Strychnia . . 130
——————— Neuralgia, in which Acetate of Morphia was successfully employed . . . . . . . 131
BARKHAUSEN, Dr. on various modes of treating Delirium Tremens . 84
BARTH, Dr. case of Hydrophobia from Cold . . . . 81
BAYNHAM, J. M. case of Retroverted Uterus, treated by Puncture of that Organ . . . . . . . . . . 507
BEGBIE, Dr. case of Abscess communicating with the Caput Coli . 265
BEHR, Dr. on Absence of the Iris . . . . . . 78
BELL, CHARLES, F.R.S. Reply of "A Pupil" to some Remarks contained in the Critical Analysis on Mr. Bell's Paper on the Nerves of the Face . . . . . . . . . . 27
BELLINI, Dr. case of Vesico-vaginal Fistula cured by actual Cautery 510
BENNERSCHEIDT, M. on the Passage of Iodine into the Blood . 181
BERNDT, M. on Chlorate of Lime in Cancer Aquaticus . . 562
BIANCINI, Dr. on the Communication between the Uterine and Placental Vessels . . . . . . . . . . 452
BIGELOW, Dr. J. on the means of affording Respiration to Children in reversed Presentations . . . . . . . 178
BILLING, Dr. case of Ossification of the left Auriculo-ventricular Valves after Rheumatic Gout . . . . . . . 322
——————— Disease of the Heart after Rheumatism . 323
BLANDIN, M. successful case of Laryngotomy . . . 177
BOILEAU, Dr. J. B. on extraction of foreign Bodies from the Œsophagus, 370
BORIE, Dr. on the treatment of Epilepsy . . . . . 462
BOUDAULT, M. case of Luxation of the Ulna backwards . . 327
BOW, Dr. W. F. on the nature of Fever and of Nervous Action (review) 65
BRICHETEAU, Dr. case of Tubercles in the Lungs of an Infant . 174
——————— Quotidian Fever, caused by an Abscess near the Anus . . . . . . . . . . ib.
BRODIE, B. C. esq. on local Effects of Poisons . . . 454
BROUSSAIS, M. on sedative Action of Asparagus . . . 276
BURGESS, Mr. J. H. case of Uterine Hemorrhage . . . 115
BURNETT, Mr. GILBERT, his Botanical Lectures announced . 374
———————, on the Natural History of the Oak . 280
BURROWS, Dr. G. M. Letter from the Surrey Medical Society to . 568
——————— review of his Letter to Sir Henry Halford, Bart. . 427

CALMEIL, M. on the Sense of Touch . . . . . 358

PAGE
CHATEAUSUF, M. case of Abscess of the Prostate Gland . . 462
CHRISTISON, Dr. on Diseases apt to be mistaken for the effects of Poisons, 455
————————, review of his Treatise on Poisons . . 133, 511
CLARET, M. on Belladonna in Neuralgia . . . . . 317
CLARK, Dr. J. notice of his work on Climate . . . . 471
CONDIE, Dr. on the use of Ipecacuanha in Hæmatemesis . . 272
CONQUEST, Dr. cases of Tapping in Hydrocephalus . . . 415
COWAN, Mr. R. case of Umbilical Hernia treated by Ligature . 559
COX, J. C. F.L.S. case of severe Injury of Head . . . 110
COXE, Dr. T. R. case in which large doses of Arsenic were given in Lepra, 272
DE SOUSA FERRAS, case of Mania from Indigestion . . . 82
DIEFFENBACH, M. on introduction of Air into the Veins of a Cock . 183

EHRLICH, Dr. case of Luxation of the Cervical Vertebræ . . 477
EISSEN, Dr. E. on treatment of Gonorrhœal Ophthalmia . . 84
ENGLAND, Dr. W. review of his work on Calculous Diseases of the Kidneys . . . . . . . . . 444
EVANS, Dr. E. on Injections of Copaiva in Gonorrhœa . . 275

FAHNESTOCK, Dr. on Datura Stramonium in Retention of Urine . 367
FORSYTH, J. S. Surgeon, review of his "First Lines of Botany" . 72
FOUILLAY, M. case of extirpation of Parotid Gland . . . 370
FOURCADE, M. on dividing the Pneumo-gastric Nerves . . 77
FRARCHINA, Dr. F. case of Metastasis during Lactation . . 461

GAITSKELL, Dr. J. A. case of Delirium in which large doses of Opium were successfully employed . . . . . . . 112
GALENZOWKI, Professor, Amaurosis cured by Extraction of a diseased Tooth . . . . . . . . . . . 464
GARDNER, Mr. on Dulcamara . . . . . . . 393
GEIL, J. B. de Hydrorrhea Uteri Gravidarum . . . 459
GRAEFE, Professor, on use of Nitrate of Silver in Diseases of the Eye 275
GRANVILLE, Dr. on Protracted Gestation . . . . . 89
GRIFFIN, Dr. W. and Mr. D. on Functional Disorders of the Spinal Cord 13, 93, 207, 307, 379
GUIBOURT, M. on the use of Nutgalls as an Antidote to the Effects of Strychnia . . . . . . . . . . 276
GUTHRIE, G. J. F.R.S. Description of his Instrument for breaking Stones in the Bladder . . . . . . . . . 564
———————— his Hunterian Oration . . . . . 280
———————— Cases reported from the Royal Westminster Ophthalmic Hospital . . . . . . 47, 219, 320
———————— Lectures at the Royal College of Surgeons . 371

HALFORD, Sir HENRY, on the Prophetic Power said to occur before Death in the Καυσος, or Brain Fever . . . . . . 277
HALL, Dr. C. case of severe lacerated Wound of the Rectum and Bladder . . . . . . . . . 367
HALL, Dr. MARSHALL, review of his Researches relative to the Morbid and Curative Effects of Loss of Blood . . . 167, 226
HAMILTON, Mr. C. B. on the use of Turpentine in Incarcerated Hernia 401
HANDEY *v.* HENSON. Trial establishing the claim of General Practitioners for Remuneration for Medical *Attendance* . . . 184
HAYWARD, Dr. G. case of Paruria Inops . . . . 484
HELIS, M. on Traumatic Delirium . . . . . . 287
HEMING, G. O. esq. on Bloodletting . . . . . . 189
HERTWICH, Dr. experiments relating to Hydrophobia . . 460
HEURTELOUP, Baron, description of his Lithotritic Instruments . 488
HIGGINBOTTOM, John, M.R.C.S.L. case of Parotid Fistula cured by the concentrated Sulphuric Acid . . . . . . 26

PAGE
HILAIRE, M. G. ST. on Vision of the Mole . . . . . 88
——————— post-mortem Examination of a double Female Infant . . . . . . . . . . 89
HODGKIN, Dr. Catalogue of the Anatomical Museum at Guy's Hospital 544
HUTCHINSON, Dr. R. S. case of Intestinal Obstruction . . 123

JÖRG, Dr. on the effects of Gastritis in Infants . . . . 80
——— on Digitalis in Hydrocephalus . . . . . 83
——— on Dropsy of the Allantoid . . . . . . 275
——— on Disorganization of the Cuticle in Infants . . 265

KAHLEISS, Dr. case of Polypus of the Vagina cured by the application of Laudanum . . . . . . . . . 369
KATERBAU, Dr. on Ossification in the Placenta . . . 178
KEATE, Mr. case of Delirium Traumaticum . . . . . 503
KING, T. esq. notice of his translation of Cloquet's Anatomy . . 472

LARREY, Baron, his mode of treating Fractures . . . 465
LEE, Dr. R. case of Uterine Phlebitis, &c. . . . . . 223
——— case of Inflammation of Saphena Veins . . . 225
——— on Inflammation of Veins of the Uterus . . . 267
——— on Phlegmasia Dolens . . . . . ib.
LEHMAN, Dr. G. F. on Otitis . . . . . . . 291
LENZ, Dr. on the use of Acetate of Lead in Phthisis . . . 464
LE SAUVAGE, M. case of Loss of Sensation; motion remaining . 359
LOMBARD, M. H. C. on Pulmonary Tubercles . . . 175
LUGOL, M. on Iodine in Scrofula . . . . . . 176
LUKE, M. case of Sloughing in the Throat, with Hemorrhage; Ligature of the Carotid . . . . . . . . . . 85

MAAS, Mr. C. F. analysis of Ergot . . . . . . 371
MACFARLANE, Dr. on treatment of Prolapsus Ani . . . 557
MARSHALL, J. esq. review of his Summary of Vaccination . . 447
MARTIN, Dr. J. case of Twins united by the Umbilicus . . 453
MARX, Dr. K. F. review of his Treatise on Toxicology . . 135
MAURIN, M. Aneurism of the Carotid cured by Ligature . . 128
MAYO, HERBERT, F.R.S. cases of Scirrhus of the Breast . . 1
MEHLHAUSEN, Dr. on Ergot in Ague . . . . . . 556
MERRILL, Dr. A. P. on Cotton Dressing for Blisters . . 83
MEYER, Dr. on external use of Morphia in Hooping Cough . . 274
MONTAULT, M. H. paralysis of the Face cured by Galvanism . 463
MORGAN, Mr. W. case of a Duplex Child . . . . . 468
MORGAN, I. F.L.S. review of his Essay on the Operations of Poisonous Agents upon the living Body . . . . . . 133
MUSSEY, Dr. R. D. case of Aneurism in which both the primitive Carotids were tied . . . . . . . . . 482

OSLERE, Dr. remarks on Ergot of Rye . . . . . 84

PARRISH, Dr. JOSEPH, on Pulmonary Consumption . . . 403
PAUL, Dr. J. M. case of Paralysis in a Child . . . . 363
PEIRCE, Dr. L. case of Perforation of Stomach . . . 215
PIORRY, Dr. on the Diagnostic Appearances of the Tongue . . 454
PURQUIN, Dr. on cure of Ague by Charcoal . . . . 365

RAHLEISS, Dr. on cure of Hooping Cough . . . . . ib.
RAINES, H. J. esq. case of Bronchocele . . . . 480
RANDALL, Dr. L. case of Wound of the Heart . . . 177
RANQUE, M. on suspending the Secretion of Milk . . . 462

PAGE
Réné Bourgeois, Dr. Recovery from Vapours of Charcoal . . 495
Richardson, Professor, on Retarded Labour, &c. . . . 177
Richond de Brus, Dr. case of Spontaneous Combustion . . 360
Roberts, Dr. C. J. case of Rupture of the Stomach . . . 475
Robinson, Dr. G. N. case of Abscess in the Brain and Aneurism of the right Subclavian Artery . . . . . . . 285
Roux, M. on Removal of Diseased Joints . . . . . 368
Russell, Mr. James, on the Causes and Prevention of Premature Delivery and Death of the Fœtus . . . . . . 299

Salmon, Mr. F. review of his work on Stricture of the Rectum, &c. 434
Schirlitz, Dr. case of Delirium Tremens . . . . . 365
Scudery, Dr. L. on the Effects of Camphor in healthy Persons . 466
Serres, M. on the Influence of the Cerebellum over the Generative Faculties . . . . . . . . . 77
———— remarks on Stammering . . . . . . 79
Sewall, Dr. T. on the use of Turpentine in Strangulated Hernia . 217
Seymour, Dr. E. J. review of his Illustrations of Ovarial Diseases . 329
Simons, Dr. T. G. on the use of Pyroligneous Acid in Gangrene, &c. 418
Smith, Dr. Southwood, review of his Treatise on Fever . . 234
Steevens, Dr. W. on the Blood . . . . . . 562
Syme, J. esq. cases of Excision of the Elbow-joint . . . 421
———————— Knee-joint . . . 423
———— case of Stricture of the Rectum, &c. . . . 426
———— on Excision of the Tonsils . . . . 465

Thomson, Dr. A. T. on Counter-Irritants . . . . 195
Tiedemann, M. on the Proportion between the Nervous System and other Parts . . . . . . . . 358
Titley, Dr. J. M. on Diseases of the Genitals in the Male . . 347
Travers, B. f.r.s. on Gonorrhœa and Syphilis . . . 552
Tweedie, Dr. A. review of his Clinical Illustrations of Fever . 234
Tyrrell, Mr. on cure of Warts by Decoction of Tormentilla . 464
———— case of Retention of Urine from Stricture; local Application of Belladonna . . . . . . . . 326

Wallaw, James, Surgeon, r.n. on Convulsions of Seamen from excessive Drinking . . . . . . . . 389
Waller, C. esq. Midwifery Report . . . . . 120
Walton, Dr. case of Abscess of the Liver, and Perforation of Stomach 79
Watson, Dr. cases of Jaundice, with Disease of the Pancreas . 499
Wendt, M. J. case in which the Rudiments of a Fœtus were found in the Testicle of an Infant . . . . . . . 358
Werlitz, M. on the Efficacy of Lemon Juice in some Diseases of the Eye . . . . . . . . . . 366
Wilkinson, Mr. case in which the Urine passed through a Fistulous Opening in the Thigh . . . . . . . 371
Wood, Dr. G. B. case of Suspension of the Cerebral Functions from Irritation of the Stomach . . . . . . . 361
Wright, Dr. T. H. case of Aortal Aneurism, with Rupture into the Trachea and Œsophagus . . . . . . . 49

Yelloly, Dr. his case of Anæsthesia . . . . . 360

COLLECTANEA . . *Pages* 77, 174, 264, 358, 452, 552
INTELLIGENCE . . . 90, 183, 277, 371, 468, 562
METEOROLOGICAL REGISTER 92, 188, 284, 378, 474, 570

---

### PLATES.

Dr. Robinson's Case of Abscess in the inferior Part of the right Hemisphere of the Brain, &c.
Aneurism of the right Subclavian Artery, &c. . . . 285

J. and C. Adlard, Printers, Bartholomew Close.

